# Circ_0045714/miR-331-3p interaction affects IL-1β-evoked human articular chondrocyte injury through regulating PIK3R3 in a ceRNA regulatory cascade

**DOI:** 10.1186/s13018-021-02738-2

**Published:** 2021-10-14

**Authors:** Ran Ding, Jinsong Zhou, Jianguo Xu, Huajie Lu, Tingting Zhang, Xiong Xiang, Zhen Shi

**Affiliations:** 1 Department of Orthopedic Surgery, Wuhan General Hospital of People’s Liberation Army, Wuhan, China; 2 Department of Pain1, Hubei Provincial Hospital of Traditional Chinese Medicine, 856 Luoyu Road, Hongshan District, Wuhan City, 430070 Hubei Province China

**Keywords:** Circ_0045714, miR-331-3p, PIK3R3, IL-1β, Human articular chondrocytes, Osteoarthritis

## Abstract

**Background:**

Osteoarthritis (OA) is characterized by joint pain and joint function limitation. Hsa_circ_0045714 (circ_0045714) is a novel OA-related circular RNA. However, its repertoire remains to be further clarified in joint chondrocytes.

**Methods:**

RNA and protein expression levels and inflammatory factor levels were detected by real-time quantitative polymerase chain reaction, western blotting and enzyme-linked immunosorbent assay. Cell proliferation and apoptosis were determined by colony formation assay, cell counting kit-8 assay and apoptosis assay. Direct interaction was predicted by bioinformatics method and confirmed by dual-luciferase reporter assay.

**Results:**

Expression of circ_0045714 and phosphoinositide-3-kinase (PI3K) regulatory subunit 3 (PIK3R3) was declined, and microRNA (miR)-331-3p was promoted in knee articular cartilages and cells from OA patients, as well as interleukin (IL)-1β-challenged human articular chondrocytes (HAC) cell line. In stimulation of IL-1β, HAC cells showed a loss of colony formation ability, cell viability and expression of Bcl-2 and Collagen II, allied with an increase in apoptosis rate and levels of IL-6, IL-8 and tumor necrosis factor-α, Bcl-2-associated X protein, cleaved caspase-3, and ADAM with thrombospondin motif-5. Noticeably, overexpressing circ_0045714 and inhibiting miR-331-3p could suppress IL-1β-evoked these effects, and both were through up-regulating PIK3R3, a key gene in PI3K/AKT signaling pathway. Mechanically, circ_0045714 functioned as competing endogenous RNA (ceRNA) for miR-331-3p and further regulated expression of the downstream target gene PIK3R3.

**Conclusion:**

There was a novel circ_0045714/miR-331-3p/PIK3R3 ceRNA axis in HAC, and its inhibition might be one mechanism of HAC injury in OA.

## Introduction

Osteoarthritis (OA) is a complex multifactorial disease, and its pathology has been advanced in genetics, genomics and epigenetics [1]. Joint pain is highly prevalent in OA patients, and severe pain is the major cause for medical attention and joint replacement [2, 3]. Inflammation has been shown to be associated with the complex etiology of joint pain in OA [4]. Besides, inflammation-related genes, tumor necrosis factor (TNF)-α and interleukin (IL)-1β are independent predictors for postoperative pain development in OA patients [5].

One of the major endpoints of OA is the loss of articular cartilage, and chondrocyte is the only cell type in the cartilage [6]. Chondrocytes are activated in the early stage of OA, accompanying with the release of pro-inflammatory cytokines and matrix catabolic enzymes, such as IL-1β and a disintegrin-like and metalloproteinase with thrombospondin motif (ADAMTS)-5 [7, 8]. Moreover, IL-1β plays versatile roles in different cell types involved in OA pathology, and IL-1β-insulted chondrocytes are suitable OA cell models [9]. Noncoding RNAs are new regulatory codes in cartilage development and skeletal disorders including OA [10], as well as the apoptosis and autophagy of chondrocytes [11]. Circular RNAs (circRNAs), a novel type of noncoding RNAs, are ubiquitous, structure-stable, tissue-specific and multifunctional. In OA, circRNAs show diagnostic and therapeutic values in the pathophysiology and treatment [12]. Hsa_circ_0045714 (circ_0045714) is a novel circRNA that is differently expressed in fracture and OA [13, 14], and it might be a therapeutic target for fracture healing and functional recovery of OA-affected chondrocytes [15, 16]. However, the role and in-depth molecular mechanism of circ_0045714 in IL-1β-induced OA model in chondrocytes are undefined yet.

Phosphoinositide 3-kinase (PI3K) regulatory subunit 3 (PIK3R3) is an inhibitor of PI3K [17] in PI3K/AKT signaling pathway which is closely interwoven with the pathogenesis of OA [18, 19]. RNA interference is a cellular mechanism for post-transcriptional gene regulation mediated by small interfering RNAs (siRNAs) and microRNAs (miRNAs) [20]. SiRNAs, a double-stranded RNA molecule with about 20 nucleotides, participate in musculoskeletal disorders and tender homeostasis and healing [21, 22]. MiRNAs are small noncoding RNAs that are useful for diagnostic or management purposes in both OA and tendon injuries [23, 24]. However, RNA interference-based regulation of PIK3R is seldom clarified yet. MiRNA (miR)-331-3p is one of miRNAs that could inactivate this pathway in carcinogenesis process [25, 26]. Nevertheless, whether there is an interaction between miR-331-3p and PIK3R3 is undetermined, as well as their functions in OA progression.

In this study, we attempted to investigate the expression and role of circ_0045714, miR-331-3p and PIK3R3 in OA patients and IL-1β-insulted human articular chondrocytes (HAC), and to further confirm the underlying relationship among them.

## Materials and methods

### Cartilages and chondrocytes isolation

OA knee articular cartilage samples and healthy cartilage samples were collected from 20 OA patients with total knee replacement and 20 trauma patients with amputation, respectively. Cartilage specimens were stored at − 80 °C. OA patients were in accordance with clinical and radiological diagnostic criteria for OA, and experienced constant pain during the last three months. Trauma patients were without any arthritis. All patients were recruited from Wuhan General Hospital of People’s Liberation Army, and each participator signed informed content. This study was ratified by the Ethics committee of this hospital.

Cartilages were minced into very small pieces and soaked into DMEM/F-12 medium (M23250; R&D systems, Minneapolis, MN, USA) containing 0.1% trypsin (B81210; R&D systems) and 0.2% Collagenase II (MX1002-100MG; MKBio, Shanghai, China) at 37 °C for 8 h. Afterward, undigested tissues were removed using 40 mm filter, and cells were harvested by centrifugation. Isolated cells were cultured in DMEM/F-12 medium (R&D systems) supplemented with 10% fetal bovine serum (FBS, S11150H; R&D systems) and 1% penicillin–streptomycin (B21210; R&D systems). Complete medium was changed every three days and cells in 2 passage were harvested for use.

### Cell culture and transfection

The immortalized HAC cell line (CHON-001; CRL-2846; ATCC, Manassas, VA, USA) was cultured in DMEM (M22650; R&D systems) containing 0.1 mg/ml G418 disulfate salt (4131; R&D systems) and 10% FBS (R&D systems). HAC cells were used to transiently transfect with exogenous nucleotides or vectors using Lipofectamine 3000 (Invitrogen, Carlsbad, CA, USA) for 36 h. The oligonucleotides were circ_0045714-siRNA (si-circ_0045714), PIK3R3-siRNA (si-PIK3R3), negative control (NC)-siRNA (si-NC), miR-331-3p mimic, miR-NC mimic, miR-331-3p inhibitor (anti-miR-331-3p), and miR-NC inhibitor (anti-miR-NC). The vectors were empty pCD5-ciR vector (GENESEED, Guangzhou, China), recombinant pCD5-ciR-circ_0045714 vector, pmiR-Reporter vector (Promega, Madison, WI, USA) expressing circ_0045714 containing the wild type (WT) or mutant type (MUT) of miR-331-3p response elements, and pmiR-Reporter vector (Promega, Madison, WI, USA) expressing PIK3R3 3’UTR containing WT or MUT of miR-331-3p response elements. Single and co-transfection models were performed per the instructions, and transfected cells at 36 h were harvested for further assays.

### OA cell model induced by IL-1β

Transfected and un-transfected HAC cells in 80% confluence were starved in serum-free medium for 4 h and then replaced with complete medium added with 10 ng/mL IL-1β (Amyjet Scientific, Wuhan, China) for 24 h. HAC cells without transfection or IL-1β treatment were served as control.

### Real-time quantitative polymerase chain reaction (RT-qPCR)

RNA was isolated with TRIzol reagent (Life Technologies, Carlsbad, CA, USA) and reversely transcribed into cDNA with SuperScript II first-strand synthesis system (Invitrogen) for RT-qPCR. SYBR Green Mix (Qiagen, Germany) and StepOne™ Real-Time PCR System (Applied Biosystems, Carlsbad, CA, USA) were adopted for the PCR amplification and melting curve analysis. 2^−ΔΔCT^ method was used to evaluate RNA expression level, and internal parameters glyceraldehyde-3-phosphate dehydrogenase (GAPDH) and U6 were used to correct RNA expression. Primers for circ_0045714, unk zinc finger (UNK), miR-331-3p and PIK3R3 are shown in Table 1.

**Table 1:** qPCR primers

	Primers (5′–3′)
circ_0045714 (135nt)	Forward: CCATGTCCAAACGTCAAGCA
	Reverse: GTGGTTCTGTGCAGGAATGG
UNK (157nt)	Forward: AGCACTACACGTACCTGAAAGAAT
	Reverse: TAATTGAAGGTGCCGTCCCG
miR-331-3p (89nt)	Forward: ACACTCCAGCTGGGGCCCCTGGGCCTATC
	Reverse: CTCAACTGGTGTCGTGGAGTCGGCAATTCAGTTGAGTTCTAGGA
PIK3R3 (118nt)	Forward: GAGCTGACTGGACTTCTCCG
	Reverse: TGGTCTGCAGAGAGCGAATC
GAPDH (104nt)	Forward: GACAGTCAGCCGCATCTTCT
	Reverse: GCGCCCAATACGACCAAATC
U6 (89nt)	Forward: GCTTCGGCAGCACATATACTAAAAT
	Reverse: CGCTTCACGAATTTGCGTGTCAT

### CircRNA characterization

Actinomycin D and RNase R assays were implemented to measure the characterization of circ_0045714, comparing to its host gene UNK. Actinomycin D (2 mg/mL; Sigma-Aldrich, St. Louis, MO, USA) was added in HAC cells for 0, 4, 8, 12 and 24 h, and RNA in each timing was isolated. RNA from HAC cells was treated with 3 U/μg RNase R (GENESEED) for 30 min, and mock cells were without RNase R treatment. Expression of circ_0045714 and UNK was detected in Actinomycin D, RNase R and mock groups using RT-qPCR.

### Colony formation assay and cell viability assay

A sum of 150 cells were seeded in 12-well plate and cultured for 15 days. Eventually, single cell was cloned into a colony, and cloned colonies were dyed with crystal violet method. Number of colonies (> 30 cells/colony) was manually determined under an inverted microscope. Cell Counting Kit-8 (CCK-8; Genomeditech, Shanghai, China) was employed to monitor cell viabilities of IL-1β-treated HAC and control cells during 3 days by measuring the optical density (OD) values at 450 nm.

### Apoptosis assay

About 5 × 10^5^ cells were co-incubated with Annexin V-fluorescein isothiocyanate (FITC) and propidium iodide (PI) according to the instructions of Annexin V-FITC/PI apoptosis detection kit (Yeasen, Guangzhou, China). Using flow cytometry (FCM), stained cells and unstained cells were analyzed: live cells were Annexin V-/PI-, early apoptotic cells were Annexin V + /PI-, and late apoptotic cells and necrotic cells were Annexin V + /PI + .

### Enzyme-linked immunosorbent assay (ELISA)

The products of IL-6, IL-8 and TNF-α in the cell culture supernatants were examined by the human IL-6 ELISA kit (EK0410; Boster, Pleasanton, CA, USA), IL-8 ELISA kit (EK0413; Boster) and TNF-α ELISA kit (EK0525; Boster), respectively. Four paralleled wells were for each group. Following the assay protocols, OD absorbance was read with a microplate reader at 450 nm, and measured concentrations were determined using linear regression of OD value against the standard curve.

### Protein isolation

Cellular protein was isolated from tissues and cells using RIPA lysis buffer (Beyotime, Shanghai, China), and culture medium protein was also isolated and concentrated via ultrafiltration membrane. Protein concentration was determined depending on BCA protein assay kit (Beyotime), and western blotting was performed in the following order: electrophoresis with polyacrylamide gel, membrane transferring via electrophoresis, antibody incubation using special primary antibodies (Table 2) and horseradish peroxidase (HRP)-labeled secondary antibody. HRP signal was reacted with BeyoECL Plus kit (Beyotime), and then detected on chemiluminescence imaging system. Protein blots were analyzed on Image-pro-plus software (Media Cybernetics, Washington, USA).

**Table 2:** Primary antibodies in western blotting

Antibody	Cat. No	Dilution rate	Source
Bcl-2	A00040-2	1:500	Boster
Bax	M00183-1	1:1 000	Boster
Cleaved Casp3	AC030	1:1 000	Beyotime
PIK3R3	M06707	1:1 000	Boster
ADAMTS-5	A12210	1:100	Boster
Collagen II	PA2141	1:500	Boster
GAPDH	M00227	1:10 000	Boster

### Dual-luciferase reporter assay

HAC cells in exponential growth were co-transfected with the recombinant pmiR-Reporter vectors expressing WT/MUT-circ_0045714 or WT/MUT-PIK3R3 3’UTR and mimic of miR-NC or miR-331-3p for 36 h. Cells were lysed and subjected to dual-luciferase reporter gene assay Kit (Beyotime). Luminescence was detected by luminometer. Relative light unit of Firefly luciferase was corrected by that of Renilla luciferase.

### Statistical analysis

Data were presented in the form of mean ± standard deviation. Each experiment was carried out in triplicate. One-way or two-way analysis of variance test followed by Tukey’s post hoc test was used to determine the differences among groups on GraphPad Prism 7 (GraphPad, La Jolla, CA, USA). *P* value < 0.05 was chosen to indicate a statistical significance.

## Results

### Down-regulation of circ_0045714 was one molecular event in OA patients and IL-1β-insulted HAC cells

**Fig. 1:**
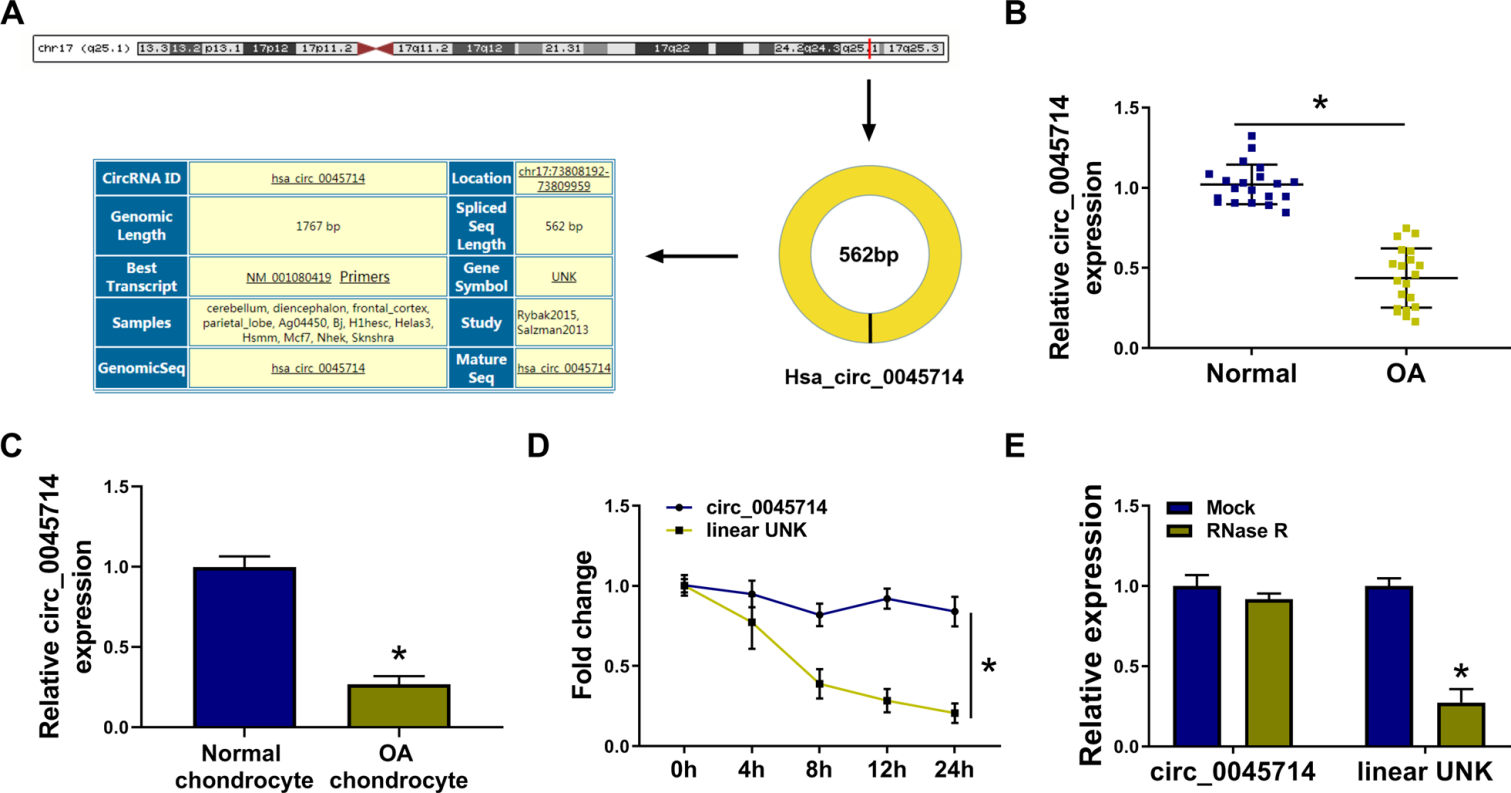
Down-regulation of circ_0045714 was one molecular event in OA. **A** The information of circ_0045714 in genome mapping was shown. **B**, **C** RNA expression was analyzed by RT-qPCR, and relative circ_0045714 expression was determined in (**B**) OA cartilages (*n* = 20) and normal cartilages (*n* = 20), and **C** OA chondrocytes and normal chondrocytes. **D**, **E** RNA expression was analyzed by RT-qPCR, and relative circ_0045714 and linear UNK expression was determined in HAC cells in actinomycin D group, RNase R group and mock group. *P < 0.05

Circ_0045714 was derived from UNK via back-splicing event (Fig. 1A), and its expression was abundantly decreased in the knee articular cartilages and chondrocytes from OA patients than normal ones (Fig. 1B, C). Besides, comparing to the host gene UNK, circ_0045714 expression in HAC cells was little affected by actinomycin D treatment during 24 h (Fig. 1D) or RNase R treatment (Fig. 1E). These data showed that circ_0045714 was aberrantly down-regulated in OA patients and IL-1β-insulted HAC.

### Hyper-expression of circ_0045714 relieved HAC apoptosis, inflammatory response and matrix degradation under IL-1β stimulation with cell proliferation inhibition

**Fig. 2:**
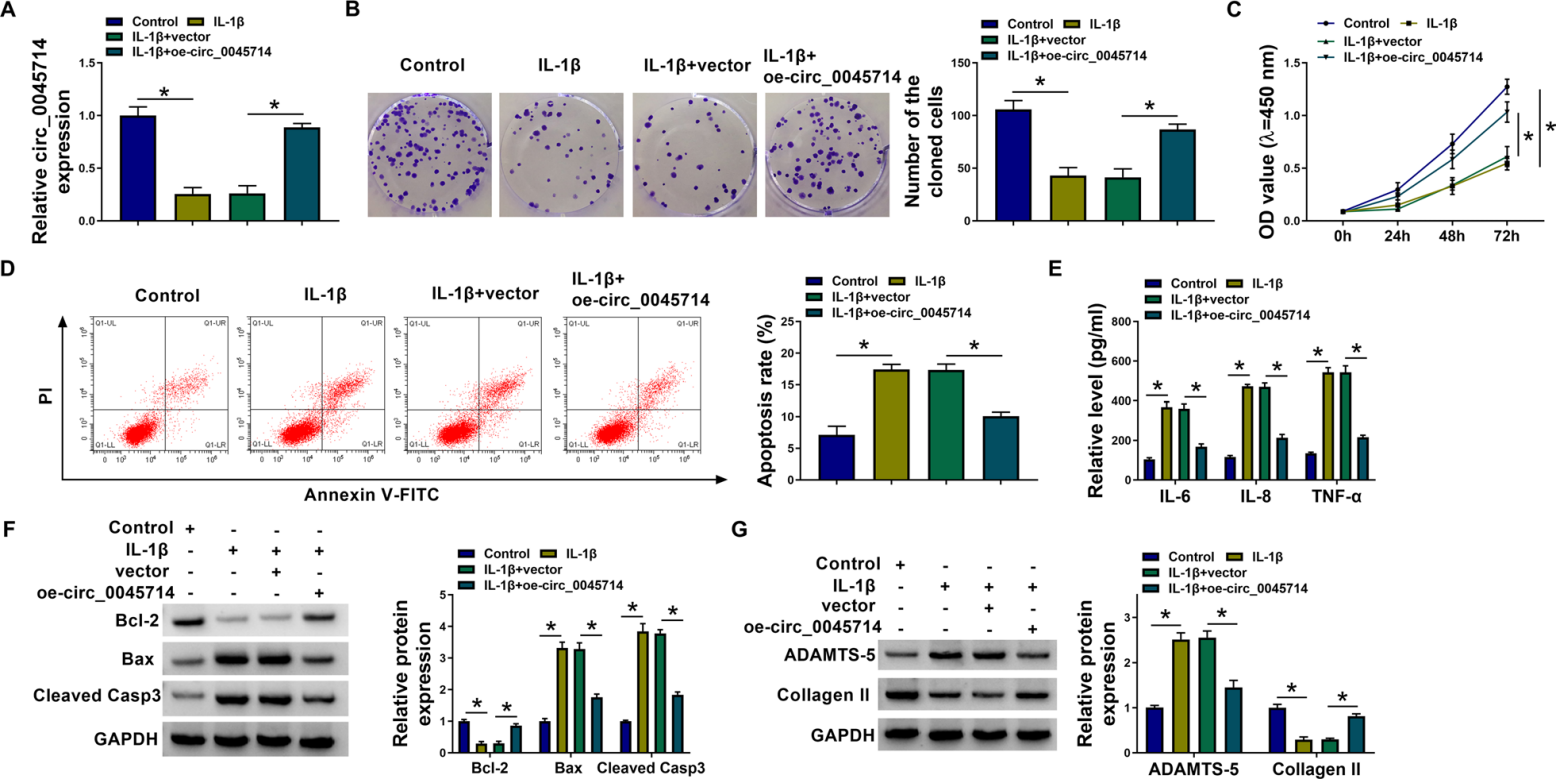
Hyper-expression of circ_0045714 relieved HAC proliferation inhibition, apoptosis, inflammatory response, and matrix degradation under IL-1β stimulation. 10 ng/mL IL-1β-treated HAC cells were pre-transfected with oe-circ_0045714 vector or empty vector. **A** RNA expression was analyzed by RT-qPCR, and relative circ_0045714 expression was determined in Control and IL-1β-treated cells. **B** Clonal survival was measured by colony formation assay, and number of the cloned cells was determined in Control and IL-1β-treated cells. **C** Cell viability was evaluated by CCK-8 assay, and OD values at 450 nm were determined in Control and IL-1β-treated cells. **D** Cell apoptosis was examined by apoptosis assay, and apoptosis rate was determined in Control and IL-1β-treated cells. **E** Secretion of inflammatory factor was detected by ELISA, and relative levels of IL-6, IL-8 and TNF-α were determined in the culture supernatants of Control and IL-1β-treated cells. **F**, **G** Protein expression was analyzed by western blotting, and relative expression of **F** Bcl-2, Bax and Cleaved in the cell lysate, and **G** Casp3, ADAMTS-5 and Collagen II in the culture supernatants was determined in Control and IL-1β-treated cells with correction with GAPDH. *P < 0.05

Genetic manipulation of circ_0045714 was carried out in OA cell model, and the expression of circ_0045714 in IL-1β-insulted HAC cells was highly promoted via oe-circ_0045714 vector transfection (Fig. 2A). A series of functional experiments were further performed, and IL-1β led to a decrease in cell proliferation in HAC cells, as evidenced by the declined number of cloned cells and cell viabilities (Fig. 2B, C). Notably, the presence of oe-circ_0045714 vector significantly improved the proliferation of IL-1β-treated HAC cells (Fig. 2B, C). In contrast, apoptosis rate and expression of apoptosis-related markers (Bax and cleaved Casp3) were sharply boosted under IL-1β stimulation, which were partially diminished due to oe-circ_0045714 vector-mediated circ_0045714 overexpression (Fig. 2D, F). IL-1β treatment evoked high production of pro-inflammatory factors (IL-6, IL-8 and TNF-α) and cartilage degradation marker (ADAMTS-5) in HAC cells, accompanying with low level of ECM component Collagen II (Fig. 2E, G), whereas IL-1β-elicited inflammation and matrix degradation in HAC cells were mitigated by reinforcing circ_0045714 (Fig. 2E, G). These results demonstrated that overexpressing circ-0045714 promoted proliferation and matrix synthesis, but suppressed apoptosis and inflammatory response in HAC under IL-1β condition.

### MiR-331-3p was up-regulated in OA and functioned as a target for circ_0045714 in HAC cells

**Fig. 3:**
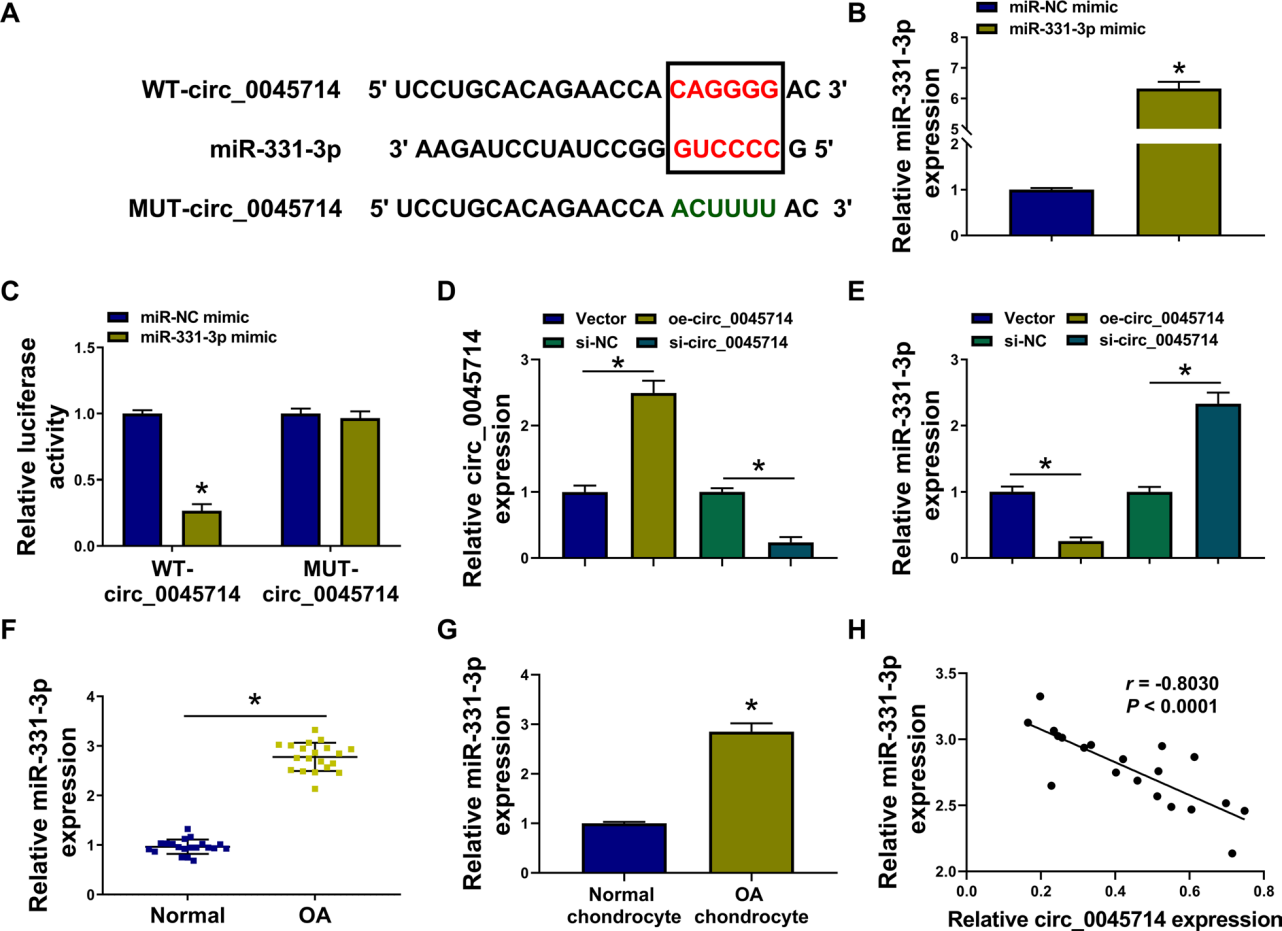
Circ_0045714 sponged miR-331-3p expression via target binding. **A** The putative miR-331-3p-binding sites and their mutations were shown in circ_0045714. **B** RNA expression was analyzed by RT-qPCR, and relative miR-331-3p expression was determined in miR-NC or miR-331-3p mimic-transfected HAC cells. **C** Luciferase activity of reporter vector was measured by dual-luciferase reporter assay, and relative luciferase activity of WT-circ_0045714 and MUT-circ_0045714 was determined in HAC cells co-transfected with miR-NC or miR-331-3p mimic. **D**, **E** RNA expression was analyzed by RT-qPCR, and relative circ_0045714 and miR-331-3p expression was determined in HAC cells transfected with oe-circ_0045714 vector, empty vector, si-circ_0045714, or si-NC. **F**, **G** RNA expression was analyzed by RT-qPCR, and relative miR-331-3p expression was determined in OA cartilages (*n* = 20) and chondrocytes and normal cartilages (*n* = 20) and chondrocytes from patients. **H** Linear correlation between two variables was analyzed using Pearson correlation test, and the correlation coefficient (*r*) between circ_0045714 and miR-331-3p in OA cartilages (*n* = 20) was determined. *P < 0.05

**Fig. 4:**
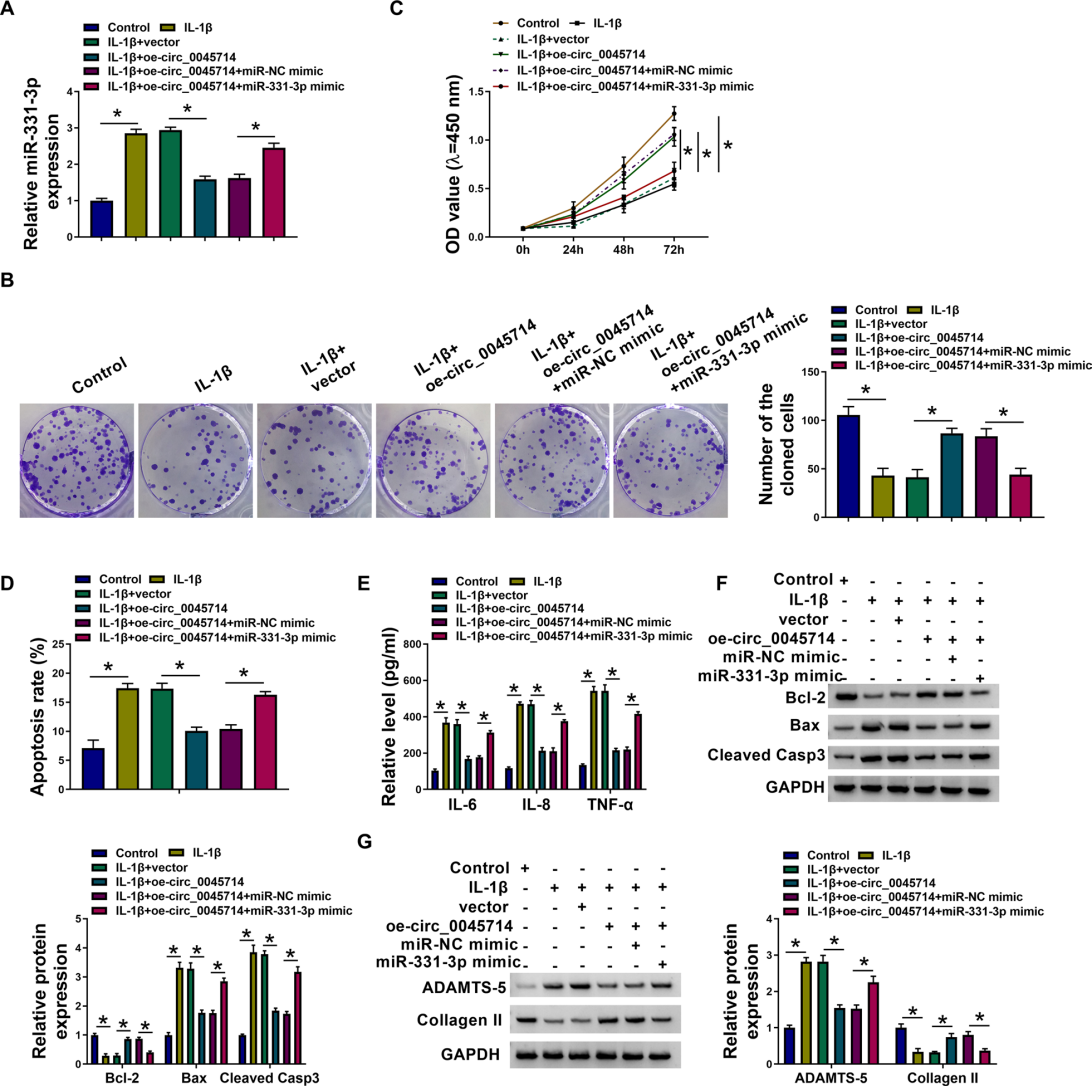
Restoring miR-331-3p counteracted the roles of circ_0045714 in IL-1β-insulted HAC. 10 ng/mL IL-1β-treated HAC cells were pre-transfected with empty vector, oe-circ_0045714 vector, or oe-circ_0045714 vector coupled with miR-NC or miR-331-3p mimic. In Control and IL-1β-treated cells, **A** relative miR-331-3p expression was detected by RT-qPCR, **B** number of the cloned cells was determined by colony formation assay, **C** OD values at 450 nm were measured by CCK-8 assay, **D** apoptosis rate was examined by apoptosis assay, **E** relative levels of IL-6, IL-8 and TNF-α in the culture supernatant were detected by ELISA, **F**, **G** relative expression of Bcl-2, Bax, Cleaved Casp3, ADAMTS-5, and Collagen II in cell lysate or cell culture supernatant was determined by western blotting with correction with GAPDH. *P < 0.05

According to the prediction result, miR-331-3p response elements in circ_0045714 were mutated for further confirmation (Fig. 3A). MiR-331-3p mimic caused overexpression of miR-331-3p in HAC cells (Fig. 3B), which then reduced the luciferase activity of WT-circ_0045714 reporter vector and left alone the mutant vector (Fig. 3C). Circ_0045714 expression was manipulated in HAC cells, and miR-331-3p level was gloomy or high when circ_0045714 was artificially overexpressed or silenced (Fig. 3D, E). In OA patients, miR-331-3p expression was elevated in the knee articular cartilages and chondrocytes (Fig. 3F, G). Moreover, Pearson correlation coefficient (*r*) between circ_0045714 and miR-331-3p levels in these 20 OA cartilages was − 0.8030 (*P* < 0.0001; Fig. 3H). And, expression level of miR-331-3p in circ_0045714-overexpressed HAC cells under IL-1β stress was also promoted by its mimic (Fig. 4A). Functionally, circ_0045714 hyper-expression-mediated enhancement of colony formation and cell viability was abated by miR-331-3p mimic co-transfection (Fig. 4B, C); allied with this was the reverse of apoptosis rate and levels of IL-6, IL-8, TNF-α, Bcl-2, Bax, Cleaved Casp3, ADAMTS-5, and Collagen II (Fig. 4D–G). These results indicated that miR-331-3p was up-regulated in human OA, and circ_0045714 functioned as a protective role in IL-1β-evoked chondrocyte injury through negatively regulating miR-331-3p via target binding.

### Exhausting miR-331-3p suppressed IL-1β-evoked HAC injury through up-regulating PIK3R3 via target binding

**Fig. 5:**
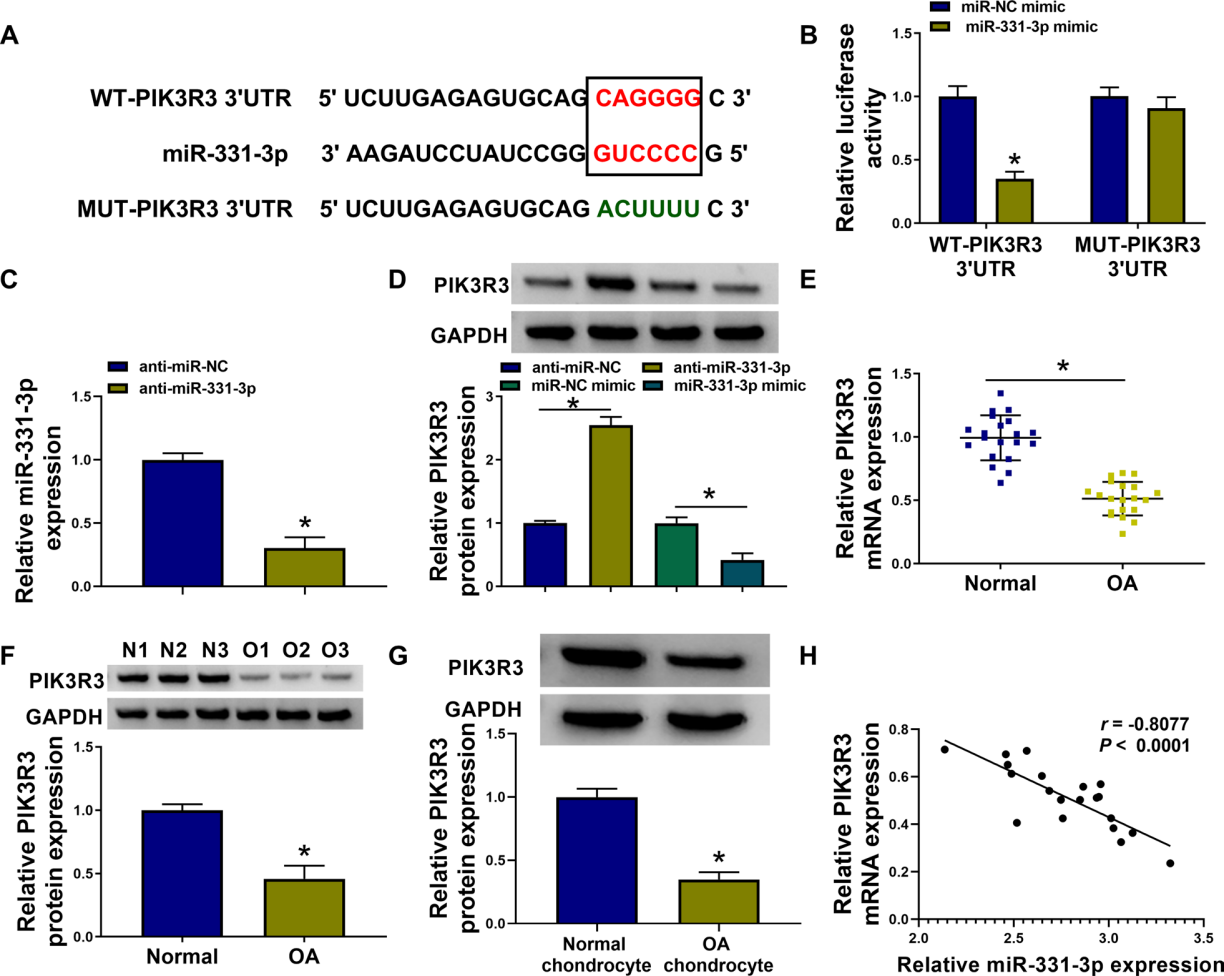
PIK3R3 was a target gene for miR-331-3p. **A** The putative miR-331-3p-binding sites and their mutations were shown in PIK3R3 3’UTR. **B** Luciferase activity of reporter vector was measured by dual-luciferase reporter assay, and relative luciferase activity of WT-PIK3R3 3’UTR and MUT-PIK3R3 3’UTR was determined in HAC cells co-transfected with miR-NC or miR-331-3p mimic. **C** RNA expression was analyzed by RT-qPCR, and relative miR-331-3p expression was determined in anti-miR-NC or anti-miR-331-3p-transfected HAC cells. **D**–**G** PIK3R3 protein and mRNA expression were respectively detected by western blotting and RT-qPCR, and its relative expression was determined in (**D**) HAC cells transfected with miR-NC, miR-331-3p, anti-miR-NC, or anti-miR-331-3p, and **E** OA cartilages (*n* = 20) and normal cartilages (*n* = 20), **F** OA cartilages (O1, O2 and O3) and normal cartilages (N1, N2 and N3), and **G** OA chondrocytes and normal chondrocytes from patients. **H** The correlation coefficient (*r*) between miR-331-3p and PIK3R3 mRNA in OA cartilages (*n* = 20) was determined using Pearson correlation test. *P < 0.05

PIK3R3 was predicted to show miR-331-3p response elements at 3’UTR (Fig. 5A). Moreover, dual-luciferase reporter assay identified the responsiveness of WT-PIK3R3 3’UTR vector to miR-331-3p overexpression mediated by the mimic (Fig. 5B). In HAC cells, protein level of PIK3R3 was facilitated or depressed in condition of miR-331-3p inhibition via inhibitor transfection or overexpression via mimic transfection (Fig. 5C, D). PIK3R3 expression at both mRNA and protein levels was lower in knee cartilages and chondrocytes isolated from OA patients than normal ones from normal patients (Fig. 5E–G). As determined by Pearson correlation test, the coefficient (*r*) between miR-331-3p and PIK3R3 mRNA levels in 20 OA cartilages was − 0.8077 (*P* < 0.0001; Fig. 5H). These results showed that PIK3R3 was abnormally down-regulated in OA through serving as downstream target gene for miR-331-3p, suggesting an interactive effect between miR-331-3p and PIK3R3 in OA progression.

**Fig. 6:**
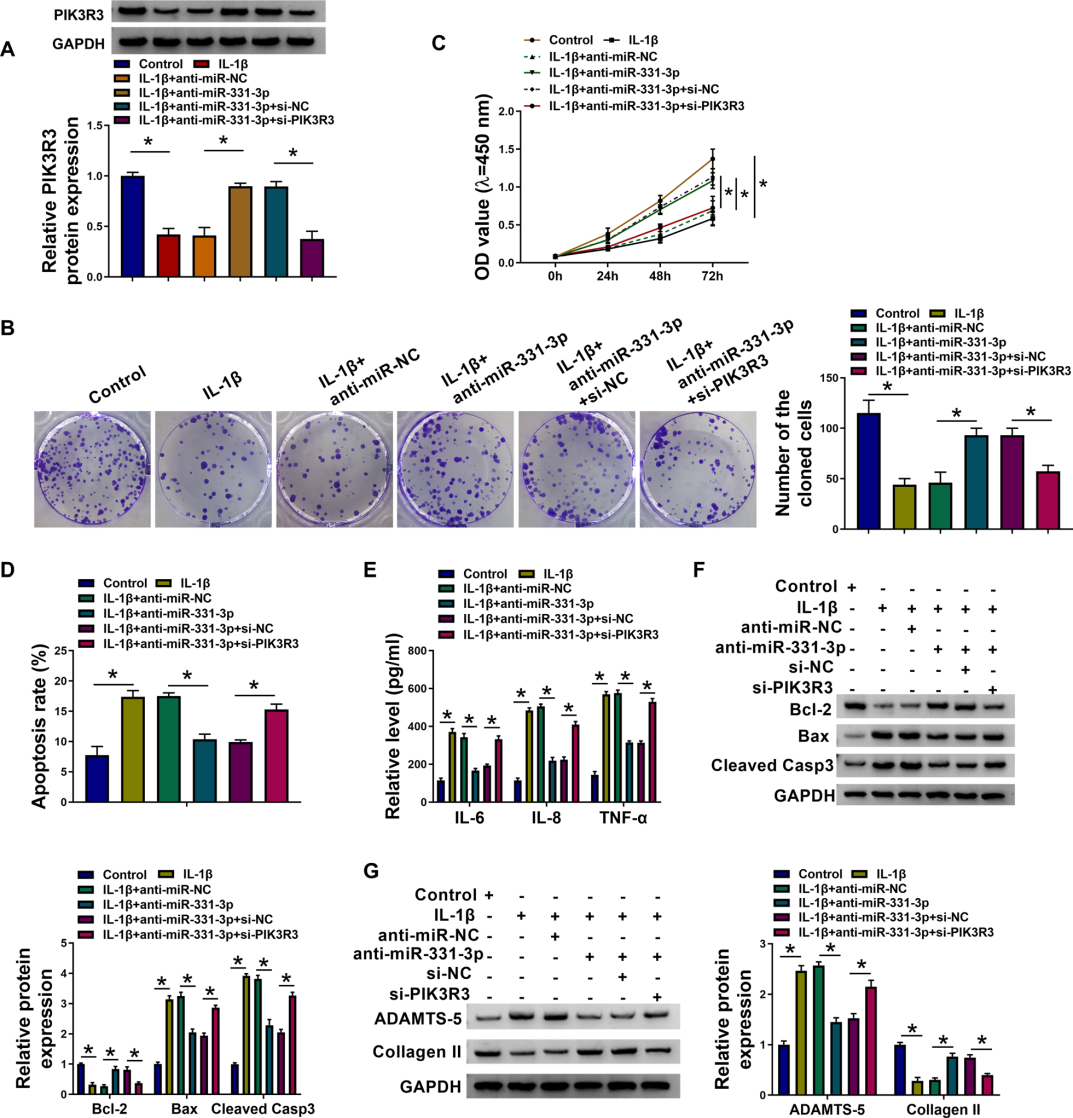
Exhausting PIK3R3 neutralized the functions of miR-331-3p inhibition in IL-1β-insulted HAC. 10 ng/mL IL-1β-treated HAC cells were pre-transfected with anti-miR-NC, anti-miR-331-3p, or anti-miR-331-3p coupled with si-NC or si-PIK3R3. In Control and IL-1β-treated cells, **A** relative PIK3R3 protein expression was detected by western blotting, **B** number of the cloned cells was determined by colony formation assay, **C** OD values at 450 nm were measured by CCK-8 assay, **D** apoptosis rate was examined by apoptosis assay, **E** relative levels of IL-6, IL-8 and TNF-α in the culture supernatant were detected by ELISA, **F**, **G** relative expression of Bcl-2, Bax, Cleaved Casp3, ADAMTS-5, and Collagen II in cell lysate or cell culture supernatant was determined by western blotting with correction with GAPDH. *P < 0.05

Functional experiments were performed in IL-1β-induced OA model by introducing anti-miR-331-3p alone or together with si-PIK3R3. As a result, PIK3R3 protein expression was inhibited in IL-1β-treated HAC cells, and endogenously silencing miR-331-3p rescued PIK3R3 level (Fig. 6A); meanwhile, PIK3R3 expression in miR-331-3p-silenced cells under IL-1β stimulation could be blocked by its siRNA (Fig. 6A). Colony number and cell viability of IL-1β-insulted HAC cells were accelerated with inhibiting miR-331-3p (Fig. 6B, C), and this acceleration was significantly weakened by inhibiting PIK3R3 (Fig. 6B, C). Contrarily, IL-1β-elicited apoptosis rate promotion and high level of IL-6, IL-8, TNF-α, Bax, and Cleaved Casp3 in HAC cells were restrained by transfecting anti-miR-331-3p, and si-PIK3R3 could cause a disinhibition for that (Fig. 6D–F). Additionally, matrix degradation (ADAMTS-5 up-regulation and Collagen II down-regulation) was induced by IL-1β in HAC cells, which was suppressed by silencing miR-331-3p (Fig. 6G); interfering PIK3R3 almost neutralized the effect of miR-331-3p inhibition on cartilage degradation under IL-1β stress (Fig. 6G). These outcomes demonstrated a counteractive role between miR-331-3p and PIK3R3 in regulating IL-1β-elicited HAC cell proliferation inhibition, apoptosis, inflammation, and ECM degradation.

### There was an interaction between circ_0045714 and PIK3R3 via miR-331-3p

**Fig. 7:**
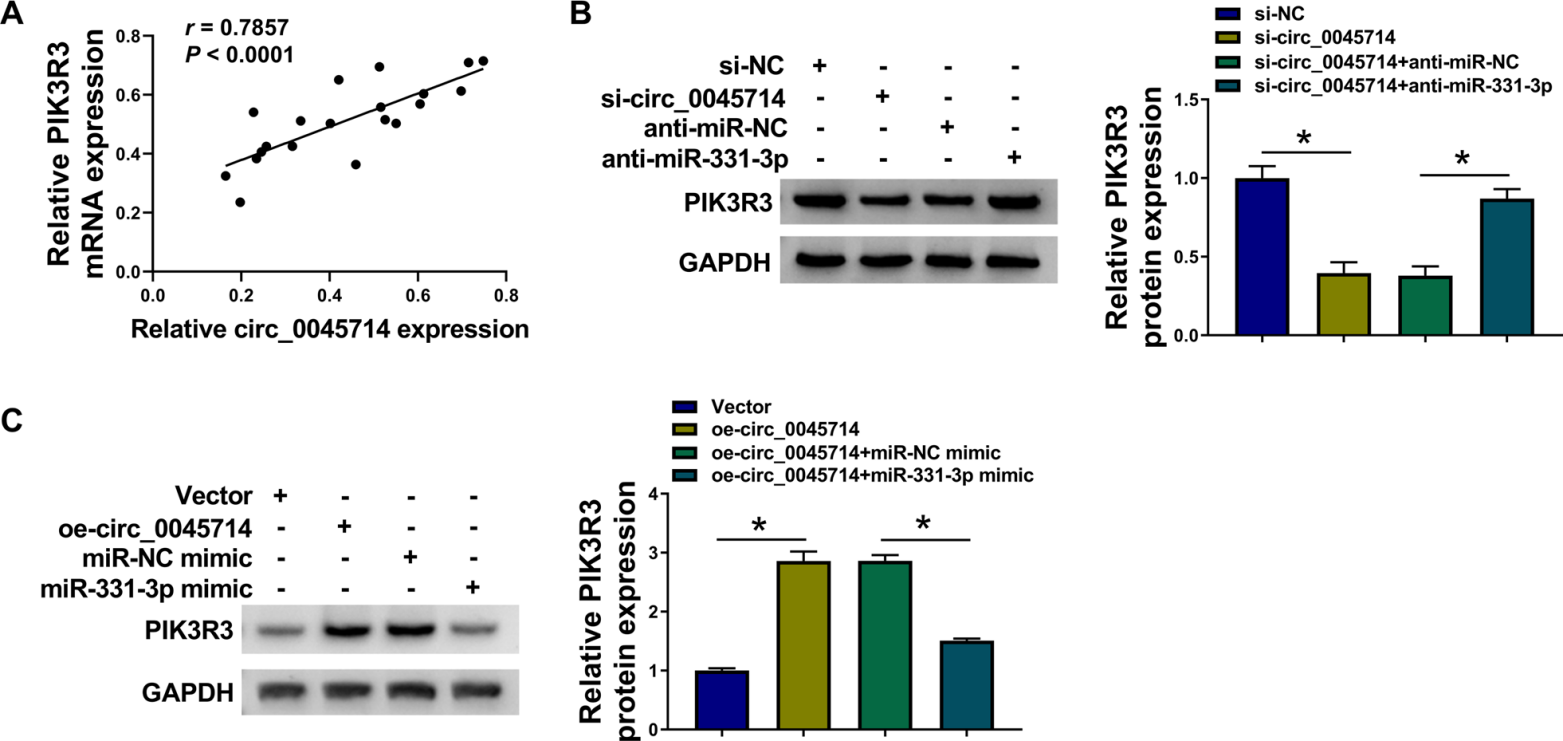
There was an interaction between circ_0045714 and PIK3R3 via miR-331-3p. **A** The correlation coefficient (*r*) between circ_0045714 and PIK3R3 mRNA in OA cartilages (*n* = 20) was determined by Pearson correlation test. **B**, **C** Western blotting detected relative PIK3R3 protein expression in HAC cells transfected with si-NC, si-circ_0045714, or si-circ_0045714 coupled with anti-miR-NC or anti-miR-331-3p, empty vector, oe-circ_0045714 vector, or oe-circ_0045714 vector coupled with miR-NC or miR-331-3p mimic. *P < 0.05

Intriguingly, there was a linear correlation between circ_0045714 and PIK3R3 mRNA levels in these 20 OA cartilages (*r* = 0.7857, *P* < 0.0001; Fig. 7A). In HAC cells, circ_0045714 mediated a positive regulatory effect on PIK3R3 protein expression, and this regulation was affected by the alteration of miR-331-3p level (Fig. 7B, C). This data showed an interaction among circ_0045714, miR-331-3p and PIK3R3 in HAC in OA.

## Discussion

CircRNAs have been proved to serve as contributors or interrupters in the pathogenesis of OA via ceRNA pathway in HAC, such as ciRS-7 [27]. In this study, we found that circ_0045714 was an interrupter in IL-1β-induced OA development, and its hyper-expression suppressed HAC apoptosis and inflammation, and improved the proliferation and ECM synthesis under IL-1β stimulation through targeting miR-331-3p and regulating PIK3R3, the inhibitor of PI3K signal.

Here, circ_0045714 expression was found to be lower in both knee cartilage tissues and cells from OA patients than normal controls, which was in favor with previous data [14, 28]. Besides, we discovered a down-regulation of circ_0045714 in IL-1β-insulted HAC, and this finding was consistent with the finding in TNF-α-induced OA model in HAC [15, 28]. Moreover, we noticed that circ_0045714 was resistant to RNase R digestion and actinomycin D (a transcriptional inhibitor) treatment. Gain-of-functional experiments revealed that circ_0045714 re-expression elevated colony formation and cell viability of IL-1β-treated HAC, and suppressed apoptosis (lower apoptosis rate and expression of Bax and cleaved Casp3), inflammatory response (higher IL-6, IL-8 and TNF-α contents), and matrix degradation (lower ADAMTS-5 and higher Collagen II), suggesting a protective role of circ_0045714 in IL-1β-induced OA in HAC. Accidently, Fang et al. [16] also proposed that circ_0045714 up-regulation could be the molecular mechanism of the relief of OA both in vitro and in vivo. Moreover, reinforcing circ_0045714 had been previously demonstrated to alleviate HAC under TNF-α stress via regulating the proliferation, apoptosis, ECM synthesis, and inflammation [15, 28]. Classically, we identified a novel ceRNA axis of circ_0045714 via targeting miR-331-3p and modulating PIK3R3.

MiR-331-3p was an inhibitor of inflammatory response in different inflammation-related diseases [29–31], including rheumatoid arthritis [32]. Here, the expression of miR-331-3p was highly induced in OA samples, including knee cartilages, *ex vivo* chondrocytes, and IL-1β-insulted HAC cells. This might be a pioneer study of miR-331-3p in human OA, and we found that inhibiting miR-331-3p might prevent HAC cells from IL-1β-evoked damages via circ_0045714/miR-331-3p/PIK3R3 axis. PIK3R3 was previously documented to be down-regulated in OA tissues and functioned as a target of miR-1236 [33]. Similarly, our data described a lower level of PIK3R3 in OA knee cartilages and *ex vivo* cartilage cells, as well as in vitro HAC under IL-1β stress. In addition, PIK3R3 was directly targeted by miR-331-3p in regulation of not only HAC cell apoptosis, but also the proliferation, inflammatory response and matrix degradation. Even though PIK3R3 was a key gene in PI3K/AKT signaling pathway [34, 35] and this way had been well-known in OA chondrocytes [36, 37], the association between PIK3R3 and chondrocyte injury was still not been elaborated yet. However, we enhanced the knowledge of RNA interference of PIK3R3 via miR-331-3p and its siRNA.

Except for PIK3R3, we also noticed that PIK3R2 and PIK3R5 were predicted to have miR-331-3p response elements according to starBase software (http://starbase.sysu.edu.cn/hsa-miR-331-3p&PIK3Rs). However, the potential target relationship between miR-331-3p and PIK3R2 or PIK3R5 was not further confirmed. By the way, PI3K/AKT pathway was the downstream of miR-155/PIK3R1 axis in dictating the cell fate of chondrocytes under IL-1β condition [38], and autophagy was affected by PI3K/AKT pathway in OA chondrocytes [27, 36]; however, PI3K/AKT pathway and autophagy were left to be further studied underlying circ_0045714/miR-331-3p/PIK3R3 axis.

## Conclusion

In conclusion, circ_0045714 and PIK3R3 were down-regulated and miR-311-3p was up-regulated in OA patients, and restoring circ_0045714 could prevent HAC against IL-1β-elicited cell apoptosis, inflammatory response and ECM degradation by regulating miR-331-3p and PIK3R3 via a ceRNA axis. Moreover, circ_0045714/miR-331-3p axis might mediate inhibition of PI3K/AKT signaling pathway via targeting PIK3R3.

## Data Availability

Not applicable.

## Abbreviations

OA: Osteoarthritis
circ_0045714: Hsa_circ_0045714
HAC: Human articular chondrocytes
PIK3R3: Phosphoinositide-3-kinase regulatory subunit 3
IL: Interleukin
TNF: Tumor necrosis factor
Bax: Bcl-2-associated X protein
ADAMTS: A disintegrin-like and metallopeptidase with thrombospondin motif
ceRNA: Competing endogenous RNA
